# Supplementary material for: Perceiving surface reflectance requires attention
Source: J Vis. 2026 Jul 23;26(7):14. doi: 10.1167/jov.26.7.14 (PMC13401182; doi:10.1167/jov.26.7.14)
Supplement: Supplement 1 [file jovi-26-7-14_s001.pdf]

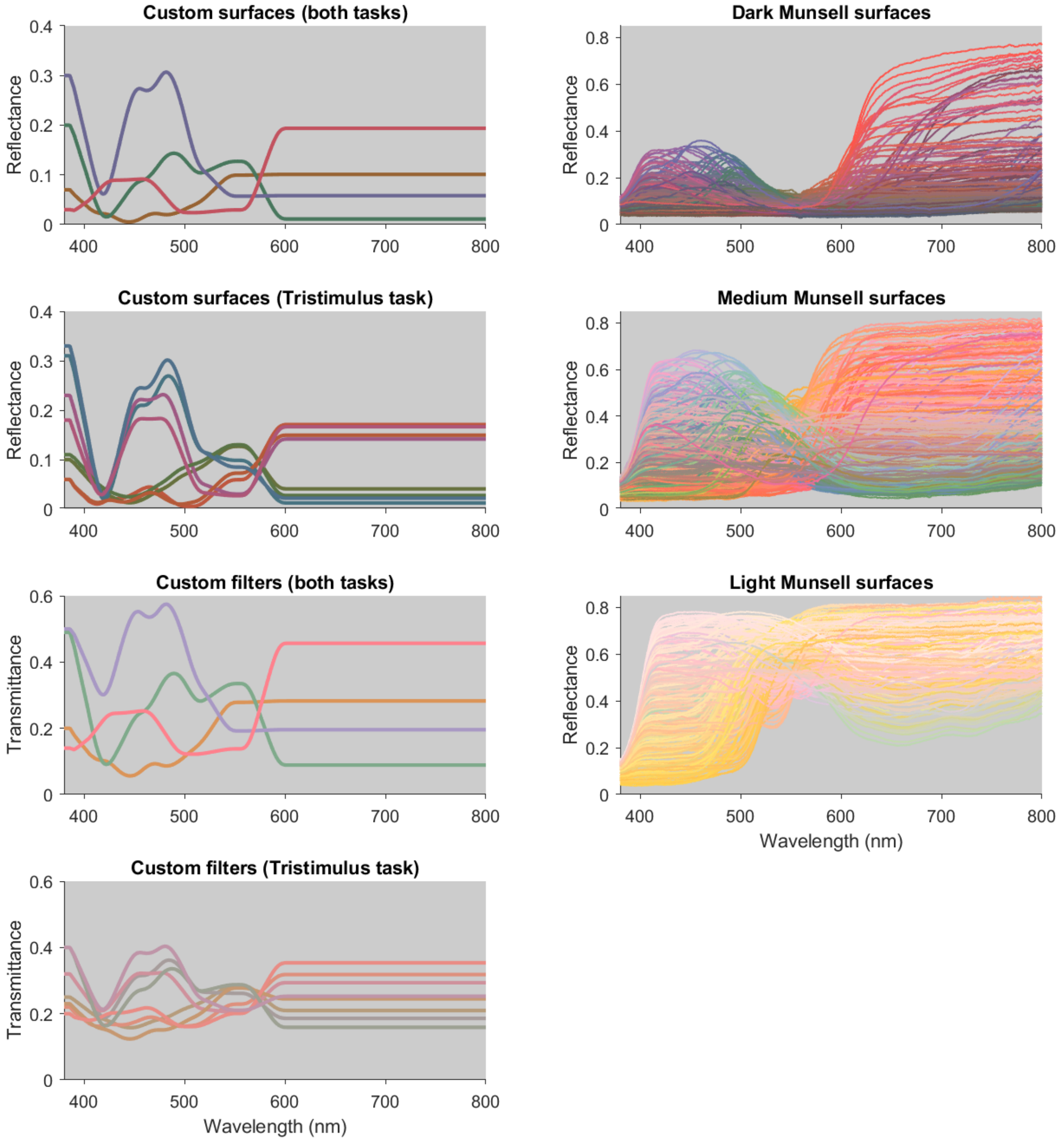

Figure S1: Surface reflectance functions and filter transmittance functions for all surfaces and filters used in the stimuli for the visual search task. In the left column, the custom surface reflectance and filter transmittance functions are shown, including all of those indicated in **Figure 1B-C**. There were 4 custom surfaces and 4 custom filters used in both the surface reflectance and tristimulus value search. To produce additional tristimulus matches, in the tristimulus value search there were a further 8 custom surface and 8 custom filters. In the right column, the Munsell surface reflectance functions are shown, divided into the three groups of values (dark, medium and light), that were assigned to all background surfaces. In each case, the functions are plotted in a colour approximating their appearance under a white illuminant (for surfaces) or when placed in front of white light (for filters).
